# Effectiveness of thoracic kinesio taping on respiratory function and muscle strength in patients with chronic obstructive pulmonary disease: A protocol of randomized, double-blind placebo-controlled trial

**DOI:** 10.1097/MD.0000000000025269

**Published:** 2021-04-09

**Authors:** Renfeng Zeng, Ke Tian, Zhike Xiao

**Affiliations:** Pulmonary and Critical Care Medicine, Affiliated Nanhua Hospital, University of South China, HengYang, China.

**Keywords:** chronic obstructive pulmonary disease, double-blind, kinesio taping, protocol, randomized

## Abstract

**Background:**

To our knowledge, only 1 study has investigated the effects of kinesio taping (KT) on pulmonary function and functional capacity of patients with chronic obstructive pulmonary disease (COPD). Therefore, there is still a lack of high-quality evidence to prove the effectiveness of KT for COPD patients. Our purpose was to investigate the effect of KT on respiratory function and muscle strength in the COPD patients who were in stable condition.

**Methods:**

This research project has been received ethical approval from the Medical Research and Ethics Committee in Affiliated Nanhua Hospital, University of South China. This work is a part of a comprehensive research project to assess and provide intervention that potentially improves respiratory function and quality of life among patients with COPD. Participants recruited into the study need to fulfill the following criteria: clinical diagnosis of COPD and symptoms indicative of exacerbation; spontaneous breathing on hospital admission; and physiotherapy since the first day of hospitalization. Patients will be assigned at random to the COPD medical treatment + KT (Group 1), or the COPD medical treatment alone (Group 2). The outcome measures are pulmonary function and respiratory muscle strength. The level of statistical significance is set as *P* < .05.

**Results:**

This protocol will provide a reliable theoretical basis for the following research.

**Conclusions:**

It was hypothesized that thoracic KT could significantly change pulmonary function and functional capacity in patients with COPD.

**Trial registration:**

This study protocol was registered in Research Registry (researchregistry6632).

## 1 Introduction

Chronic obstructive pulmonary disease (COPD) is a chronic inflammatory disorder caused by the inhalation of tobacco smoke or other irritants. It is characterized by progressive deterioration in pulmonary function and persistent airway inflammation. Its symptoms include difficulty in breathing, coughing, mucus (sputum) production, and wheezing.^[1]^ Patients with COPD usually exhibit an upper chest breathing pattern and dyspnea on exertion. The disease also presents extrapulmonary manifestations such as skeletal muscle dysfunction, which is an important prognostic factor that justifies exercise intolerance in these patients.^[2]^

Several treatment strategies for COPD are provided in the current literature, which includes limited bed rest, pharmacological therapy, Oxygen therapy, and physiotherapy. Physiotherapy interventions, such as breathing exercises and early mobilization, aim to restore or maintain muscle function during an acute episode of COPD.^[3–5]^ Physical therapists often use these strategies to relieve dyspnea, improve thoracoabdominal coordination, and enhance functional capacity in patients with COPD exacerbations.^[6]^

A relatively new treatment method for COPD is kinesio taping (KT), which is being widely used as a relatively novel band-aid method to reduce the pain of musculoskeletal disorders. KT is an elastic bonding material containing high tensile capacity, which ensures the free movement of the application area without the need of drugs or chemicals. KT can be extended to 140% of the original length, providing a good range of motion in comparison with other types of tape.^[7,8]^ Studies have shown that KT improves blood and lymph circulation, mitigates pain, adjusts joints, and relives muscle tension. Although the effect of KT on pain is unclear, KT may provide afferent stimuli that promote pain inhibition mechanisms and pain relief.^[9,10]^ Depending on the proposed effects, we speculated that if KT were applied to thorax, it could have some beneficial effects to restore respiratory muscle function and to reduce hyperinflation, thereby resulting in increased functional capacity for patients with COPD.

To our knowledge, only 1 study has investigated the effects of KT on pulmonary function and functional capacity of patients with COPD.^[11]^ Therefore, there is still a lack of high-quality evidence to prove the effectiveness of KT for COPD patients. In this study, our purpose was to investigate the effect of KT on respiratory function and muscle strength in the COPD patients who were in stable condition. It was hypothesized that thoracic KT could significantly change pulmonary function and functional capacity in patients with COPD.

## 2 Materials and methods

### 2.1 Ethical approval

This work is a part of a comprehensive research project to assess and provide intervention that potentially improves respiratory function and quality of life among patients with COPD. This research project has been registered in the research registry (with number: researchregistry6632) and received ethical approval from the Medical Research and Ethics Committee in Affiliated Nanhua Hospital, University of South China (no. Tzey3208523). After the introductory briefing, the acceptance to take the presession questionnaire implied the participant's consent to participate in the study.

### 2.2 Population

Participants recruited into the study need to fulfill the following criteria: clinical diagnosis of COPD and symptoms indicative of exacerbation; spontaneous breathing on hospital admission; and physiotherapy since the first day of hospitalization. The exclusion criteria are as follows: patients who are uncooperative, those have previous thoracic surgery, thoracic deformities or any condition that make it difficult to apply the KT, and those with reports of allergic skin reactions to the use of adhesive bandages, plasters, or other adhesive materials.

### 2.3 Random and blind

**Figure 1:**
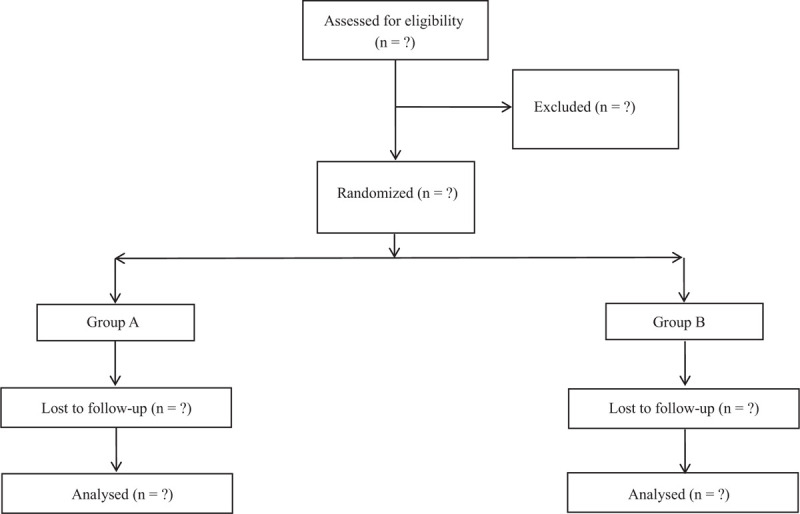
PRISMA flow diagram describing the selection process for relevant clinical trials used in this meta-analysis.

Before starting the study, a randomization list is produced using software-generated randomized numbers; the randomization depends on random blocks of 10. Patients will not know to which group they are assigned or which treatment they will be offered. Patients will be assigned at random to the COPD medical treatment + KT (Group 1), or the COPD medical treatment alone (Group 2). Participants are enrolled by the research assistant. After baseline examination, all patients will be given a full explanation of the treatment protocol and will be required to sign a written informed consent for study participation and for publication of the results. All the data collectors, doctors, statistical analysts, as well as result assessors are not aware of grouping assignment (Fig. 1).

### 2.4 Intervention protocol

In Group 1, KinesioTex Gold is used for KT application. Patients are seated on a chair. When the patients are at the end of the expiration, it will be applied bilaterally on the fifth to sixth and ninth to tenth intercostal muscles transversally and on the anterior and posterior axillary line longitudinally with 50% tension. To stimulate the muscle and support the function, the stimulation technique is usually applied from the origin to the insertion of the muscle with the recommended stretching of 25 to 50%. KT is changed on every fifth day (for a total of 3 times and 15 days) by the same certified physiatrist who has previously attended the KT training course. The patients are instructed not to take the tapes off at any time but they are allowed to take showers. The researcher, who is blind to the type of treatment, completes posttreatment (15th day) evaluations for each patient and recorded the data.

### 2.5 Outcome measure

The outcome measures are pulmonary function and respiratory muscle strength. Pulmonary function was measured using a spirometer. To perform the measurement, subjects were instructed to sit on an armless chair with back support while their knees flexed to 90° and nostrils occluded by a nose clip. Then they performed a maximal inspiration followed by a forced expiration, exhaling as much air as possible 3 seconds. This maneuver was repeated 3 times, and the highest scores were recorded for analyses. Respiratory muscle strength will be measured as maximum inspiratory pressure (MIP) and maximum expiratory pressure (MEP), using an analogic manovacuometer. The assessments are performed in accordance with American Thoracic Society/European Respiratory Society standards (2002). The participants are assessed in the seated position. The MIP maneuver begins near residual volume, and the MEP maneuver begins near total lung capacity. The maximum score of 3 maneuvers varying by less than 20% will be recorded.

### 2.6 Statistical analysis

All analyses will be performed using SPSS for Windows, version 16, and GraphPad InStat. Analysis of variance is used for comparing mean values of patient's age, weight, height, and body mass index. For nonparametric measures, differences between baseline and posttreatment scores for each group are computed by the Wilcoxon signed ranks test. The difference between each treatment group is performed by the Kruskal–Wallis test. The level of statistical significance is set as *P* < .05.

## 3 Discussion

The purpose of medical treatment of COPD is to reduce the frequency and severity of the symptoms and to improve the functional capacity and the quality of life. To our knowledge, only 1 study has investigated the effects of KT on pulmonary function and functional capacity of patients with COPD. Therefore, there is still a lack of high-quality evidence to prove the effectiveness of KT for COPD patients. In this study, our purpose was to investigate the effect of KT on respiratory function and muscle strength in the COPD patients who were in stable condition. It was hypothesized that thoracic KT could significantly change pulmonary function and functional capacity in patients with COPD. In our protocol, a total of 120 subjects will be recruited.

## Author contributions

**Conceptualization:** Renfeng Zeng, Ke Tian.

**Data curation:** Renfeng Zeng, Ke Tian.

**Formal analysis:** Renfeng Zeng, Ke Tian.

**Funding acquisition:** Zhike Xiao.

**Investigation:** Renfeng Zeng, Ke Tian.

**Methodology:** Renfeng Zeng, Ke Tian, Zhike Xiao.

**Project administration:** Zhike Xiao.

**Resources:** Zhike Xiao.

**Software:** Renfeng Zeng, Ke Tian.

**Supervision:** Zhike Xiao.

**Validation:** Ke Tian.

**Writing – original draft:** Renfeng Zeng, Ke Tian.

**Writing – review & editing:** Zhike Xiao.
